# Vaginal lubricants in the couple trying-to-conceive: Assessing healthcare professional recommendations and effect on in vitro sperm function

**DOI:** 10.1371/journal.pone.0209950

**Published:** 2019-05-14

**Authors:** Scott C. Mackenzie, Steven A. Gellatly

**Affiliations:** School of Medicine, University of Dundee, Dundee, Scotland; University of Porto, PORTUGAL

## Abstract

Vaginal lubricants are commonly used by couples trying-to-conceive. However, most vaginal lubricants are sperm toxic and therefore should not be used by couples trying-to-conceive. Despite this, lubricant sperm toxicity is insufficiently reported and guidance for healthcare professionals (HCPs) is absent. In this study, lubricant-related practices of fertility-based HCPs in Scotland were sampled via an online survey. Lubricants identified as being utilised in the fertility setting were subsequently incubated with prepared sperm samples to establish effects on sperm motility. HCP recommendations (n = 32) on lubricant use were varied although knowledge related to sperm toxicity was generally poor. HCPs infrequently asked about lubricant use and were unaware of guidance in this area. Aquagel, the only prescribed lubricant identified in this study, reduced sperm progressive motility to 49% of control after 10 minutes, even at concentrations as low as 5%. Vitality testing suggested the deterioration in progressive motility with Aquagel was not as a result of cell death. Conversely, Pré Vaginal Lubricant, a ‘sperm-safe’ lubricant, did not significantly affect any markers of sperm function assessed. Development of clinical guidance in this area is recommended to ensure HCPs deliver informed advice as lubricant use in couples trying-to-conceive may inadvertently contribute to delay in conception.

## Introduction

Vaginal lubricants are commonly used by couples to manage vaginal dryness and make intercourse more comfortable [1,2]. Although lubricants are of widespread availability and popularity, current regulation does not require lubricant packaging to clearly state the impact a lubricant may have on sperm function or the natural fertility process. Couples trying-to-conceive (TTC) represent a subgroup in which vaginal dryness is common [3]. A recent UK survey of over 1000 women of reproductive age actively TTC found that ~10% of participants reported active lubricant use at the time of the survey, a proportion significantly larger than those experiencing vaginal dryness (3%) [4]. In light of such widespread use of vaginal lubricants, it is essential that patients make appropriately informed choices. However, doctor-patient communication barriers combined with limited evidence and insufficient guidance for healthcare professionals (HCPs) regarding appropriate lubricant use makes this a challenging field in which to deliver advice.

A number of studies over the last five decades have demonstrated the detrimental effects of different lubricants on sperm function [2,5–18]. Motility, progressive motility and vitality are the most commonly assessed as markers of sperm function. Although variations in methodology and reporting make quantitative review unsuitable, the in vitro data collected is in general agreement that most commercially available lubricants negatively impact sperm function, albeit variably.

The search for a sperm-safe lubricant dates back to the late 1950’s [19], however more recently the situation has been clarified with the United States Food and Drug Administration introducing a unique product code (‘PEB’) for personal lubricants that after sufficient testing are considered ‘gamete, fertilization and embryo compatible’ [20]. Such regulation and labelling in the US does not guarantee universal HCP or patient awareness of such issues, however strives to regulate and standardise a previously unclear marketplace. However, similar regulation does not currently exist in the United Kingdom, and lubricant use in couples TTC may negatively impact chances of conception.

## Materials and methods

### Healthcare professional survey

See S1 Appendix for full survey. The practices of HCPs were evaluated via a cross-sectional online survey. NHS ethical approval was not required for this survey as this project fell within the definition of service evaluation [21]. The survey was made using the BOS online survey tool (Available at: www.onlinesurveys.ac.uk). A pilot survey was carried out on a group of n = 10 HCPs studying a postgraduate degree in reproductive medicine to assess ease of use and readability. The survey was distributed via email to members of the Scottish Fertility Network. Participants for the survey were HCPs (doctors and nurses) working in NHS or private fertility clinics across Scotland. Although the total number of HCPs working in fertility clinics is not publicly available, the total number of IVF cycles performed per clinic is publicly available via inspection reports available from the Human Fertilization and Embryo Authority [22]. We were therefore able to estimate the total number of HCPs present in Scotland by extrapolating our knowledge of the number of HCPs present locally in Dundee Assisted Conception Unit. The most recent available inspection report was used for each clinic. Through this estimation approximately n = 120 HCPs are present in Scotland. Responses to the survey therefore represent approximately 27% of the total HCP population. The survey was available online for a period of approximately 8 weeks. Survey responses were anonymous at the point of collection and participants were not asked to disclose the identity of their clinic in the questionnaire in-order to encourage openness and honesty in their responses. Descriptive statistics were used for all other data collected. Data was processed, and figures were produced in GraphPad Prism 8.0.2 for macOS, GraphPad Software, La Jolla California USA, www.graphpad.com.

### Subjects and ethical approval

Semen samples were obtained from n = 15 healthy donors with no known fertility problems. Samples were collected by masturbation into a plastic container after 48 to 72 hours of ejaculatory abstinence. Written consent was obtained from all sperm donors and all donors were recruited in accordance with the HFEA Code of Practice (version 8) under local ethical approval (13/ES/0091) from the East of Scotland Research Ethics Service (EoSRES) REC 1.

### Sample and lubricant preparation

All samples were prepared using a discontinuous density gradient using PureCeption 40%/80% (Origio, Denmark) within 1 hour of ejaculation. Density gradient prepared sperm were used as this selects motile spermatozoa that are likely to reach the site of fertilization and is consistent with similar studies referenced herein. The 80% pellet was washed, and samples were diluted to approximately 15 million sperm/mL in Quinn’s Sperm Washing Medium (ART-1006; Origio, Denmark). In the first experiments, motile sperm fractions from n = 5 different donors were incubated for 0, 10, 30 and 60 minutes at 37°C in 10% (v/v) solutions of two lubricants: Pré Vaginal Lubricant (INGfertility, Valleyford, WA) and Aquagel (Ecolab Ltd, Leeds, UK). Aquagel is a KMJ Class: 1 lubricant. Pré Vaginal Lubricant is a FDA gamete, fertilisation, and embryo compatible personal lubricant [20]. All lubricants were in date at the time of use. Control samples were incubated for 0 and 60 minutes in medium alone and a 10% (v/v) solution of K-Y Jelly (Johnson & Johnson, Santé Beauté, France). K-Y Jelly was used as a negative control based on the numerous published accounts of its damaging effects on sperm function [5,11,14]. The 10% (v/v) lubricant dilution is reflective of the concentration used in previously published studies [2,12,14]. The range of incubation times chosen are consistent with a previously published study suggesting that the majority of fertilising sperm migrate through the cervix within 30 minutes after ejaculation [23]. In subsequent experiments, motile sperm fractions from n = 5 different donors were incubated for 10 minutes at 37°C in 10% (v/v), 5% (v/v), 1% (v/v) and 0.2% (v/v) solution of Aquagel. 10 minutes was chosen as this was the earliest point at which we had previously observed changes in progressive motility following incubation with 10% (v/v) Aquagel.

### Osmolality of lubricant preparations

100 uL of each 10% (v/v) lubricant in Quinn’s Sperm Washing Medium was added to a 1.5 mL Eppendorf tube. The osmolarity was then measured using a Löser freezing point micro-osmometer type 15/15M (ThermoFisher, Paisley, UK). Osmolarity was measured in triplicate (3 separate incubations of each lubricants in Quinn’s Sperm Washing Medium) and results expressed as mean ± SEM. These values were compared with osmolality reference values for human sperm function (see Table 1).

**Table 1:** Osmolality of human secretions.

Human secretion	Osmolality (mOsm)
Semen	320 [24]
Mid-cycle cervical mucus	250–422 [25]
Vaginal secretions	260–290 [26]

Osmolality expressed in milliosmoles.

### Motility analysis

Motility was evaluated at 37°C using computer-assisted-sperm analysis (SCA 5.1, Microoptic, Barcelona) by phase-contrast microscopy (Zeiss, Axiostar plus, Germany) equipped with a Basler A312fc digital colour camera (Microptic, SL.L., Barcelona, Spain) at x200 magnification. For microscopic assessments, duplicate wet-preparations were prepared for each experimental condition using pre-warmed 2-cell Leja chamber and 4 μL of the sample giving depth of 20 μm. At least 200 spermatozoa in at least 5 microscope fields of view were examined in each duplicate. Sperm motility was assessed under a negative phase contrast objective (x200 magnification) with the system parameter settings for these analyses being 25 frames at 25 frames per second (Hz) and particle area for detection of spermatozoa head being 2–60 μm^2^. A minimum of 20 data points was used for tracking a cell. Sperm motility was classified using a four-category scheme: rapid progressive (≥25 μm/s), slow progressive (4–24 μm/s), non-progressive (≤4 μm/s), and immotile [27]. Motility data was presented as progressive motility (sum of rapid progressive motility and slow progressive motility). For each duplicate motility assessment, an average value was calculated only when the difference between the replicate values were within acceptable range, as determined using Table 2.1 in the World Health Organisation laboratory manual for the examination and processing of human semen [28]. If the replicate value was out with the acceptable range, this data point was excluded from further analysis.

### Sperm vitality

Sperm vitality was measured in n = 5 motile sperm fractions from n = 5 different donors after 60 minutes incubation with 10% (v/v) Aquagel, 10% (v/v) K-Y Jelly and 10% (v/v) Pré Vaginal Lubricant using the hypo-osmotic swelling test (HOST). For the non-lubricant control, the equivalent amount of Quinn’s Sperm Washing Medium was added. HOST was performed using 1 mL of a hypo-osmotic solution (0.75 mM fructose (Sigma-Aldrich, Poole, UK), 0.75 mM sodium citrate (Sigma-Aldrich, Poole, UK) in distilled H_2_O; osmolality of 150 mOsm/kg) for incubation of 100 μL of prepared spermatozoa for 30 min at 37°C. 200 spermatozoa were analysed using a phase contrast microscope (x400 magnification) as curled (viable sperm) or not curled (non-viable sperm), according to World Health Organisation [28].

### Statistical analysis

Statistical significance for motility and vitality data was calculated using a one-way ANOVA based on the assumption that the data was approximately normally distributed. When one-way ANOVA resulted in a p-value < predefined α error (α = 0.05), a post-hoc pairwise Tukey’s HSD Test was carried out to determine statistical significance between individual groups. A p-value <0.05 was considered statistically significant. As motility data for Aquagel and Pré Vaginal Lubricant was recorded at intermediate time points (10 and 30 minutes) which control data was not recorded at, paired t-tests compared to 0 minutes were used to determine statistical significance at these points where a p-value <0.05 was considered statistically significant. All statistical analyses were performed using GraphPad Prism 8.0.2 for macOS, GraphPad Software, La Jolla California USA, www.graphpad.com.

## Results

### Healthcare professional survey

A total of n = 32 complete responses, comprising n = 19 nurses, n = 12 doctors and n = 1 respondent who did not indicate their profession, was available for analysis. The respondent who did not indicate their profession completed all other sections of the survey and was therefore included in our analysis. Enquiring about lubricant use in couples TTC was uncommon among respondents with 85% (n = 28) of HCPs *rarely* or *never* asking about lubricant in the medical history. However, most HCPs (81% (n = 26)) would not recommend use of a lubricant in couples TTC. HCPs were asked to explain why they would or would not recommend a lubricant for a couple TTC and n = 19 responses were received. Combatting uncomfortable intercourse or avoidance of intercourse secondary to vaginal dryness (n = 9) were the most common reasons for recommending a lubricant and many HCPs stated that they would only recommend a lubricant for these purposes. One respondent stated that they had never discussed this issue with patients before and one stated they had never considered lubricants as a possible cause of infertility. One respondent acknowledged the sensitive nature of this aspect of medical history taking, explaining that vaginal dryness is explored as an issue if patients identify it when asked about problems with intercourse.

Specific lubricants recommended by HCPs included Astroglide, Conceive Plus, Pre-seed, K-Y Jelly, Sliquid Oceanics Natural Lube, and ‘water-based lubricants’. Only 9% (n = 3) of respondents had prescribed a lubricant for a patient in the past. Aquagel (Ecolab Ltd) was the only lubricant that was identified as having been previously prescribed. HCPs were asked if they were aware of any guidance, either local or national, addressing lubricant use in couples TTC. All respondents surveyed (n = 32) stated that they were unaware of any guidance.

**Fig 1:**
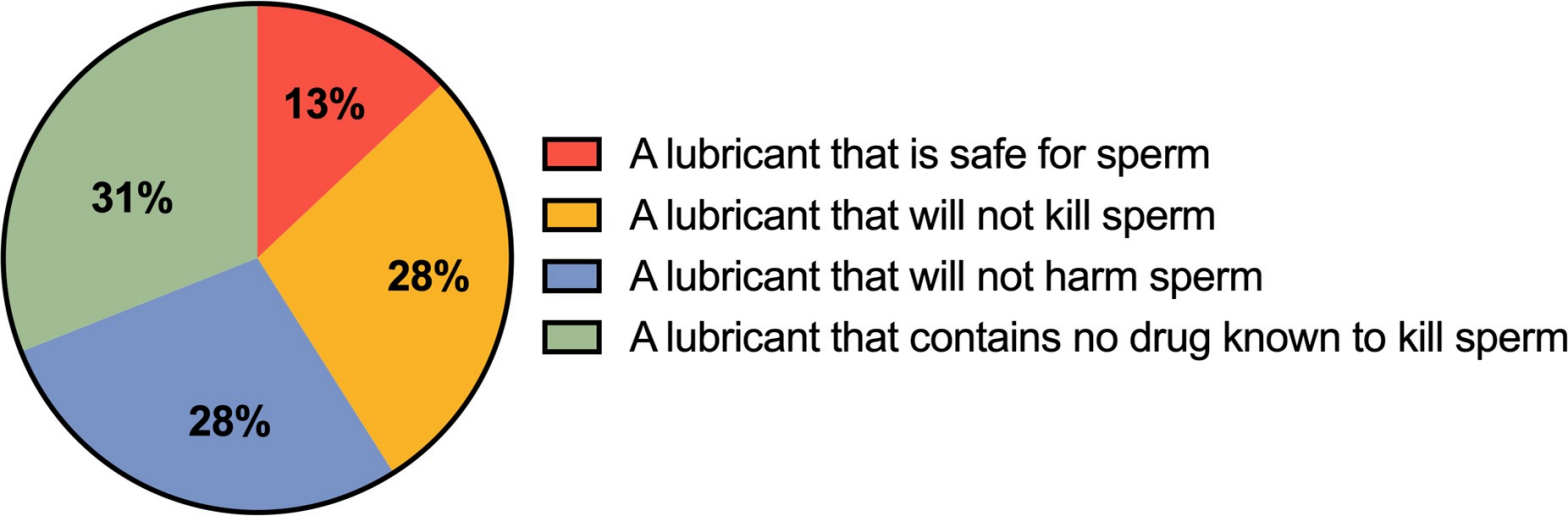
Which of the following best describes a lubricant with the classification ‘non-spermicidal’?. Pie chart representing n = 32 responses. The correct answer is ‘a lubricant that contains no drug known to kill sperm’.

HCPs were asked to what extent they agreed with the following statement: *‘Lubricants marketed for fertility patients can have an impact on sperm function’*. The majority of respondents (66% (n = 21)) chose a neutral stance opting to ‘*neither agree nor disagree*’. HCPs were asked to identify the best definition, out of a possible 4, that described a non-spermicidal lubricant. Only 31% (n = 10) of respondents identified the correct response: a non-spermicidal lubricant contains no drug known to kill sperm (see Fig 1).

### Osmolarity of lubricant preparations

The osmolarity in the 10% (v/v) lubricant solutions are presented in Table 2. The osmolarity of all lubricants apart from K-Y Jelly were within limits that could be considered physiological with reference to values expressed in Table 1.

**Table 2:** Osmolality of lubricant preparations.

Lubricant preparation (10% v/v)	Osmolality (mOsm ±SEM)
SAGE Quinn’s Sperm Washing Medium	284.3 (± 1.2)
Pré Vaginal Lubricant	287.0 (± 0.6)
K-Y Jelly	1301.7 (± 2.7)
Aquagel	359.7 (± 4.2)

Osmolality expressed in milliosmoles ± standard error of mean.

### The in vitro effect of lubricants on sperm function

**Fig 2:**
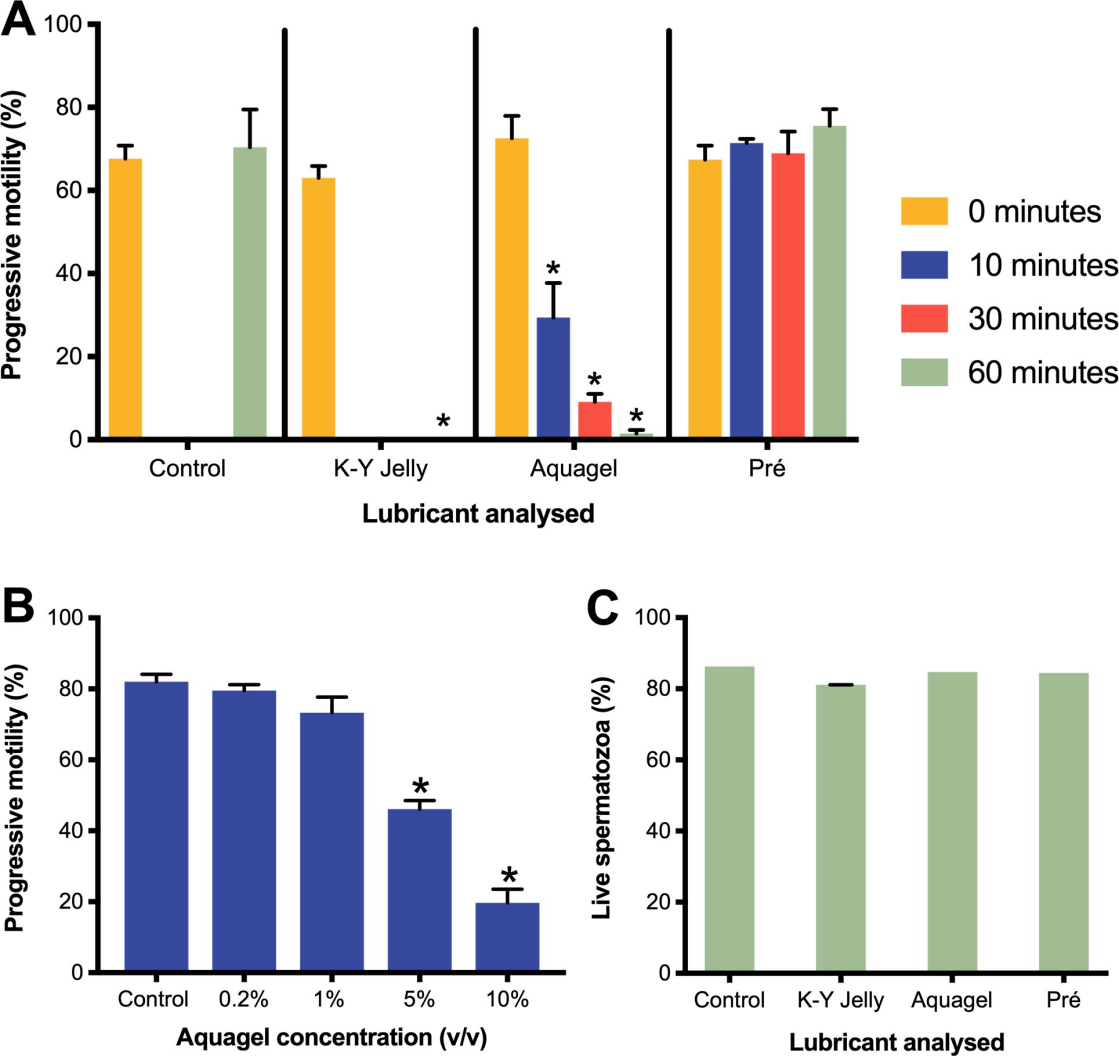
Sperm progressive motility and vitality results summary. Graphs A, B and C represent data from n = 5 prepared samples from n = 5 different donors per experiment. A represents percentage progressive motility after 60 minutes exposure to 10% (v/v) lubricant. B represents percentage progressive motility after 10 minutes exposure to Aquagel at varied concentration. C represents vitality after 60 minutes 10% (v/v) Aquagel exposure. * represents p≤0.01. In graphs A, B, and C error bars represent standard error. Error bars are negligibly small for control, Aquagel and Pré Vaginal Lubricant in graph C and are not displayed. Pré refers to Pré Vaginal Lubricant.

See Fig 2 for results summary. 10% (v/v) Aquagel significantly reduced progressive motility (PM) to 2% of control at 60 minutes (p<0.01). Significant reductions in PM after 10% (v/v) Aquagel exposure were also observed at 10 (p<0.01) and 30 minutes (p<0.01). Incubation with 10% (v/v) K-Y Jelly significantly reduced PM to negligible levels at 60 minutes (p<0.01). Incubation with 10% (v/v) Pré Vaginal Lubricant did not significantly change PM compared to control at any time assessed. Vitality analysis after 60 minutes incubation with 10% (v/v) Aquagel and 10% (v/v) K-Y Jelly showed no difference in vitality between 10% (v/v) Aquagel or 10% (v/v) K-Y Jelly and control, suggesting PM decreases were not secondary to cell death (see Fig 2C). 60 minutes incubation with 10% (v/v) Pré Vaginal Lubricant did not result in a decrease in vitality compared to control.

Aquagel was subsequently incubated with samples at 10% (v/v), 5% (v/v), 1% (v/v) and 0.2% (v/v), with PM assessed at 10 minutes (post-lubricant addition). 10% (v/v) Aquagel significantly reduced PM to 25.6% of control at 10 minutes (p<0.01). 5% (v/v) Aquagel significantly reduced PM to 58% of control at 10 minutes (p<0.01). No significant changes to PM were observed at 1% (v/v) or 0.2% (v/v).

## Discussion

It is unclear if HCP avoidance of lubricant use in history-taking is due to a lack of awareness or HCPs sharing the personal embarrassment, perceived unacceptability or perceived unimportance reported by patients with regard to general sexual health issues [29,30]. A common approach across HCPs was to discuss the topic if patients identified it was an issue upon general sexual health questioning but given that women often expect leadership from HCPs in raising sexual health issues, this approach may be inappropriate leaving many patients who are open to advice without appropriate guidance [29]. It is therefore suggested that HCPs consider integrating querying lubricant-use in their routine history taking in the fertility setting. Additionally, educational intervention has been shown to significantly increase HCP awareness of the importance of discussing sexual health with patients and may be an appropriate method to increase lubricant-related questioning among HCPs [31]. An important distinction is that the term ‘non-spermicidal’, commonly used to classify lubricants, is a drug classification specifying that a product does not contain a spermicidal drug. As discussed by Mortimer et al. [32], this classification does not mean a product is sperm-safe or that it will not harm or kill sperm. This misconception was common among surveyed HCPs and is likely present among patients.

Although we captured approximately 27% of the Scottish HCP population, we recommend this number is interpreted with caution as it is based on an estimate total number of HCPs working in Scottish fertility clinics as previously mentioned. Therefore, at this stage we are unable to reliably comment on how representative this data is for Scotland. However, we feel it is unlikely that there is a significant proportion of unsampled HCPs that adopt a structured approach to lubricant recommendations, particularly given the lack of national guidance identified in this study. Regulation of lubricants appropriate for couples TTC is present in the United States and this is not the case in Scotland [20]. However, lubricant related practices of HCPs in the United States have never been studied and it is therefore unclear if such regulation directly translates into improved HCP awareness.

Conceive Plus and Pre-seed were both recommended by a HCP in this study and these lubricants are PEB ‘gamete, fertilization and embryo compatible’ and therefore appropriate for use in couples TTC [20]. Aquagel is a multipurpose lubricant available for NHS prescription as listed in the NHS Drug Tariff Part IX [33]. This study found that Aquagel decreased progressive motility in a time- and concentration-dependant manner, even at concentrations as low as 5% (v/v). Compared to lubricants analysed in Anderson et al. [2], Agarwal et al. [14] and Sandhu et al. [18], Aquagel is one of the most sperm toxic ‘non-spermicidal’ lubricants currently available in the marketplace, however the cause(s) of this toxicity are yet to be established. In agreement with past findings [2,13,14], K-Y Jelly was also found to be detrimental for sperm motility. In this study we used discontinuous density gradient prepared spermatozoa as these are the sperm that are likely to reach the site of fertilisation in the oviduct. Kutteh et al. [13] and Anderson et al. [2] measured sperm motility on discontinuous density gradient prepared sperm, whereas Agarwal et al. [14] used raw semen and a decrease in sperm motility was seen in sperm fractions of both prepared and raw groups. Unfortunately the effect of Aquagel in raw semen is yet to be investigated, but given the previous findings of K-Y Jelly mentioned above we would expect similar results. Anderson et al. [2] suggested that the high osmolality of K-Y Jelly may cause damage to tail membranes resulting in impaired motility, however the full molecular mechanism(s) of this effect awaits further investigation.

Unfortunately, we only report progressive motility results following incubation with different lubricants. Future work should involve tests of sperm function (sperm kinematics, ability to undergo the acrosome reaction) and DNA integrity in the presence of the Aquagel. Agarwal et al. [14] found K-Y Jelly to significantly decrease DNA integrity compared to control in raw semen, whereas Mowat et al. [17] found no difference, however Mowat et al. used discontinuous density gradient prepared spermatozoa rather than raw semen which may explain the discrepancy in the result. Pré Vaginal Lubricant has previously been shown not to negatively impact DNA or chromatin integrity [20]. The reversibility of the effect of vaginal lubricants on sperm function remains unclear and this has not been assessed in the literature base to date. Further experiments should investigate whether sperm motility is able to recover post-lubricant exposure.

Appropriate lubricant advice is ultimately quick, easy and free and may help couples achieve natural pregnancy. Vaginal lubricants should be avoided when TTC unless specifically indicated to manage or prevent sexual dysfunction and this should be the message relayed by HCPs to patients. When indicated, Pré Vaginal Lubricant is an appropriate choice and this study supports its FDA classification as a ‘gamete, fertilization and embryo compatible’ lubricant. Aquagel should not be used or prescribed in couples TTC to manage vaginal dryness. This study did not assess all the lubricants identified by HCPs and further work could aim to address this. However, vaginal lubricants should be assumed to be not sperm safe and therefore not recommended or prescribed in those TTC unless robust evidence is present suggesting the contrary. It must be emphasised that although many vaginal lubricants significantly impair motility they cannot be relied upon as contraceptives and patients should be made aware of this.

Development of guidelines and patient information in this area is required to improve both HCP and patient understanding of the issues presented. These guidelines should also be shared with doctors working in general practice aiming to identify problematic lubricant usage as early in a couples ‘fertility journey’ as possible. Until such guidance and information is developed, clinics are encouraged to discuss these issues and develop a unified approach to lubricant recommendations to inform patient usage where appropriate.

## Supporting information

S1 Appendix Healthcare professional survey. (PDF)
