# Supplementary material for: Vaginal lubricants in the couple trying-to-conceive: Assessing healthcare professional recommendations and effect on in vitro sperm function
Source: PLoS One. 2019 May 14;14(5):e0209950. doi: 10.1371/journal.pone.0209950 (PMC6516666; doi:10.1371/journal.pone.0209950)
Supplement: S1 Appendix — (PDF) [file pone.0209950.s001.pdf]

Healthcare Professional Survey

Q1. Please indicate your role.

A. Doctor
B. Nurse

Q2. How often do you bring up the issue of vaginal dryness when you see couples who are trying to conceive?

A. Always
B. Often
C. Sometimes
D. Rarely
E. Never

Q3. How often do you ask about lubricant use in couples trying to conceive?

A. Always
B. Often
C. Sometimes
D. Rarely
E. Never

Q4. Would you recommend a vaginal lubricant for a couple trying to conceive?

A. Yes
B. No

Q4a. Please provide a reason for your choice.

Q5. Which lubricant would you recommend for a couple trying to conceive?

A. I would not recommend a lubricant
B. Any lubricant
C. Any lubricant marketed as nonspermicidal

Q5a. If you would recommend a lubricant, please provide further details.

Q6. Are you aware of any local or national guidance on lubricant recommendations for couples trying to conceive? (please circle one)

A. Yes
B. No

Q6a. If yes, please specify below.

Q7. Have you ever prescribed a particular lubricant for a couple trying to conceive?

A. Yes
B. No

Q7a. If yes, which lubricant have you previously prescribed? (detail below)

Q8. To what extent do you agree with the following statement: "Lubricants marketed for fertility patients can have an impact on sperm function".

A. Strongly agree
B. Agree
C. Neither agree nor disagree
D. Disagree
E. Strongly disagree

Q9. Which of the following best describes a lubricant with the classification "nonspermicidal"?

A. A lubricant that is safe for sperm
B. A lubricant that will not kill sperm
C. A lubricant that will not harm sperm
D. A lubricant that contains no drug known to kill sperm

Thank you for participating.
